# [18F]FLT and [18F]FDG PET for Non-invasive Treatment Monitoring of the Nicotinamide Phosphoribosyltransferase Inhibitor APO866 in Human Xenografts

**DOI:** 10.1371/journal.pone.0053410

**Published:** 2013-01-04

**Authors:** Mette Munk Jensen, Kamille Dumong Erichsen, Camilla Bardram Johnbeck, Fredrik Björkling, Jacob Madsen, Michael Bzorek, Peter Buhl Jensen, Liselotte Højgaard, Maxwell Sehested, Andreas Kjær

**Affiliations:** 1 Cluster for Molecular Imaging, Faculty of Health and Medical Sciences, University of Copenhagen, Copenhagen, Denmark; 2 Department of Clinical Physiology, Nuclear Medicine and PET, Rigshospitalet, Copenhagen, Denmark; 3 TopoTarget A/S, Symbion Science Park, Copenhagen, Denmark; 4 Department of Pathology, Næstved Hospital, Næstved, Denmark; The University of Chicago, United States of America

## Abstract

**Introduction:**

APO866 is a new anti-tumor compound inhibiting nicotinamide phosphoribosyltransferase (NAMPT). APO866 has an anti-tumor effect in several pre-clinical tumor models and is currently in several clinical phase II studies. 3′-deoxy-3′-[18F]fluorothymidine ([18F]FLT) is a tracer used to assess cell proliferation *in vivo*. The aim of this study was non-invasively to study effect of APO866 treatment on [18F]FLT and 2-deoxy-2-[18F]fluoro-D-glucose ([18F]FDG) uptake.

**Methods:**

*In vivo* uptake of [18F]FLT and [18F]FDG in human ovary cancer xenografts in mice (A2780) was studied at various time points after APO866 treatment. Baseline [18F]FLT or [18F]FDG scans were made before treatment and repeated after 24 hours, 48 hours and 7 days. Tumor volume was followed with computed tomography (CT). Tracer uptake was quantified using small animal PET/CT. One hour after iv injection of tracer, static PET scans were performed. Imaging results were compared with Ki67 immunohistochemistry.

**Results:**

Tumors treated with APO866 had volumes that were 114% (24 h), 128% (48 h) and 130% (Day 7) relative to baseline volumes at Day 0. In the control group tumor volumes were 118% (24 h), 145% (48 h) and 339% (Day 7) relative to baseline volumes Day 0. Tumor volume between the treatment and control group was significantly different at Day 7 (P = 0.001). Compared to baseline, [18F]FLT SUVmax was significantly different at 24 h (P<0.001), 48 h (P<0.001) and Day 7 (P<0.001) in the APO866 group. Compared to baseline, [18F]FDG SUVmax was significantly different at Day 7 (P = 0.005) in the APO866 group.

**Conclusions:**

APO866 treatment caused a significant decrease in [18F]FLT uptake 24 and 48 hours after treatment initiation. The early reductions in tumor cell proliferation preceded decrease in tumor volume. The results show the possibility to use [18F]FLT and [18F]FDG to image treatment effect early following treatment with APO866 in future clinical studies.

## Introduction

During development of new anti-cancer drugs, methods to assess early treatment effect and discriminate between responders and non-responders are widely needed. The use of positron emission tomography (PET) to discriminate between responders and non-responders early following treatment initiation is desirable. The PET technique allows for non-invasive characterization of biological functionality in tumor tissue. 3′-deoxy-3′-[18F]fluorothymidine ([18F]FLT) is used to assess cell proliferation *in vivo* by PET, by measuring the activity of thymidine kinase 1 (TK1) which is up-regulated in the S-phase of cell cycle [1]–[6]. Uptake of [18F]FLT has in several pre-clinical models been shown to decrease following treatment with various chemotherapeutics much earlier than a response in tumor volume is evident [7]–[16]. However, changes in [18F]FLT uptake following treatment are variable and dependent upon mode of action for the different chemotherapeutic drugs [7], [9]–[11], [13].

APO866 is a new anti-tumor compound which inhibits nicotinamide phosphoribosyltransferase (NAMPT), an enzyme involved in the biosynthesis of NAD. APO866 inhibits biosynthesis of cellular NAD from niacinamide by inhibiting NAMPT. Reduction in cellular NAD levels leads to ATP depletion and apoptosis [17], [18]. Cancer cells have a high NAD turn over compared with normal cells. NAD is an important cofactor for poly(ADP-ribose) polymerase 1 (PARP1) which is involved in DNA repair and therefore cancer cells are more sensitive to NAMPT inhibition than normal cells. APO866 has anti-tumor effect in many tumor cells *in vitro* and several pre-clinical human xenograft tumor models [19], [20]. A clinical phase I study with APO866 has already been conducted [21], and APO866 is currently in several clinical phase II studies.

APO866 treatment decreases cellular ATP content [17], [19] and ATP is a cofactor for TK1 [22]. We therefore hypothesized that [18F]FLT uptake would be influenced by APO866 treatment due to the decrease in ATP and possibly a change in tracer uptake could be used to predict treatment response at an early stage.

2-deoxy-2-[18F]fluoro-D-glucose ([18F]FDG) has in several pre-clinical and clinical studies been tested for its ability to assess early effect of chemotherapeutics; however, with variable results. When new anti-cancer drugs are brought into clinical phase II and III studies, effective methods to discriminate between responders and non-responders are of great value. The aim of this study was therefore non-invasively to study the effect of APO866 treatment on [18F]FLT and [18F]FDG uptake and to evaluate if [18F]FLT or [18F]FDG PET could be used to monitor early effects of APO866 treatment.

## Materials and Methods

### Tumor Model

Animal care and all experimental procedures were performed under the approval of the Danish Animal Welfare Council (2006/561–1124). Female NMRI (Naval Medical Research Institute) nude mice (6–8 weeks old) were acquired from Taconic Europe (Lille Skensved, Denmark) and allowed to acclimatize for one week in the animal facility before any intervention was initiated. The human ovarian carcinoma cell line A2780 [23] (a gift from R. Ozols, Fox Chase Cancer Center Philadelphia, PA, USA) was used. A total of 10^7^ cells in 100 µL medium mixed with 100 µL Matrixgel™ Basement Membrane Matrix (BD Biosciences, San Jose, CA, USA) were injected subcutaneously into the left and right flank respectively during anesthesia with 1∶1 v/v mixture of Hypnorm® (Janssen Pharmaceutica, Beerse, Belgium) and Dormicum® (Roche, Basel, Switzerland). The cell line has been tested free of mycoplasma. Cells were cultured in RPMI (Roswell Park Memorial Institute) medium 1640+ GlutaMAX (Invitrogen, Carlsbad, CA, USA) supplemented with 10% fetal calf serum (Biological Industries, Israel) and 1% penicillin-streptomycin (Invitrogen) in 5% CO_2_ at 37°C.

### Experimental Design

**Figure 1:**
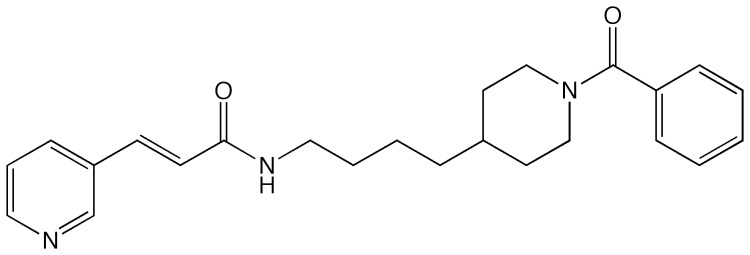
Chemical structure of the nicotinamide phosphoribosyltransferase inhibitor APO866.

Mice were divided into groups (n = 6–12 tumors per group) receiving either APO866 or vehicle treatment and [18F]FLT or [18F]FDG scans. Treatment was started at day 14–16 after implantation of tumor cells, where tumor volumes were on average 265 mm^3^. Mice in the treatment groups received APO866 (figure 1) in saline/PBS with 2% HP-β-CD and 9% propylene glycol at a dose of 15 mg/kg IP twice daily for 7 days. This is the maximum tolerated dose of APO866 and inhibits growth of the A2780 xenograft tumor model [20]. The control groups received vehicle twice daily for 7 days. The mice were scanned at baseline before treatment start with [18F]FLT or [18F]FDG and subsequently [18F]FLT or [18F]FDG scans were repeated at 24 h (Day 1), 48 h (Day 2) and 168 h (Day 7) hours after start of treatment with either APO866 or vehicle. Tumor volume was followed by microCT during the experiments [24]–[26]. In addition to the microCT measurement of tumor volume, tumor sizes were followed by external caliper measurements where tumor volume was calculated using the modified ellipsoidal formula ½(length*width^2^). At Day 7 tumors were excised and transferred to −80°C for Ki67 immunohistochemistry analysis. All tumors were weighted and tumor weight was correlated with both microCT and caliper determined tumor volume.

### Synthesis of [18F]FLT and [18F]FDG

[18F]FLT was synthesized using 3-N-Boc-1-[5-O-(4,4′-dimethoxytrityl)-3-O-nosyl-2-deoxy-b-D-lyxofuranosyl]thymine as precursor on a GE TracerLab MX Synthesizer as described earlier [7]. All reagents and [18F]FLT cassettes were purchased from ABX (Radeberg, Germany). [18F]FDG was acquired from daily productions for clinical use (Rigshospitalet, Copenhagen, Denmark).

### microPET and microCT Imaging

Mice were injected i.v. with 10.3±1.4 (mean±SD) MBq [18F]FDG or 9.5±0.9 (mean±SD) MBq [18F]FLT. Mice were fasted overnight before each [18F]FDG scan [27]. One hour after tracer injection mice were anaesthetized with 3% sevoflurane (Abbott Scandinavia AB, Solna, Sweden) mixed with 35% O_2_ in N_2_ and fixed on a bed in presence of three fiducial markers allowing fusion of PET and CT pictures. A 10 min PET scan was acquired using a MicroPET Focus 120 (Siemens Medical Solutions, Malvern, PA, USA). After data acquisition, PET data were arranged into sinograms and subsequently reconstructed with the maximum a posteriori (MAP) reconstruction algorithm. The pixel size was 0.866×0.866×0.796 mm and in the center field of view the resolution was 1.4 mm full-width-at-half-maximum. The images were not corrected for attenuation or scatter.

Following the microPET scan, a microCT scan was acquired with a MicroCAT® II system (Siemens Medical Solutions). A 7 minute and 10 seconds CT scan was performed with parameter settings: 360 rotation steps, tube voltage 60 kV, tube current 500 µA, binning 4 and exposure time 310 ms. The pixel size was 0.091×0.091×0.091 mm.

PET and microCT images were fused in the Inveon software (Siemens Medical Solutions). Before fusion region of interests (ROIs) were drawn on the CT pictures manually by qualitative assessment covering the whole tumors and subsequently tumor volume and tracer uptake, assessed by standard uptake values (SUV) mean and maximum, were generated by summation of voxels within the tomographic planes. SUV was defined as (C_T_*W)/D_inj_, where C_T_ is radioactivity in the tissue, W is weight of the animal and D_inj_ is injected dose. SUVmean gives information of the mean concentration of tracer and SUVmax is a measure of the voxel within the ROI which have the highest tracer concentration. For tumor volume measurements ROIs were drawn manually by delineation of the tumor boundary by qualitative assessment in several of the coronal planes on the microCT images. Summation of voxels from all the planes generated the tumor volume.

### Ki67 Immunohistochemistry

After freezing at −80°C tissue samples were fixed in 4% neutral buffered formalin and subsequently embedded in paraffin. The anti-human Ki-67 clone MIB-1 (Dako cat.no F7268/FITC conjugated mouse antibody) was used. The reactions were detected using standard polymer technique with few modifications. Negative controls were performed by omission of primary antibody. Sections were dewaxed in xylene, rehydrated and pre-treated before immunostaining. The slides were incubated with MIB-1 1:100 diluted in Power Block solution (BioGenex,cat. no. HK085-5K) for 30 min at room temperature. The sections were rinsed in TBST (3×5 minutes) and incubated with rabbit anti-FITC (Invitrogen, cat. no. A6413 1:250) for 20 min at RT. The reactions were detected with Super Sensitive polymer-HRP reagent (Biogenex, cat. no. QD410D-XCX) and visualised with DAB ImmPACT Substrate chromogen solution (Vector laboratories cat. co. SK-4105) following the manufactures instructions. Finally, slides were counterstained with Mayers haematoxylin.

The level of Ki67 present in the samples was measured as mean of three hot spots on one slide from each tumor. Hotspots were defined as areas with the highest Ki67 intensity and were identified by scanning the entire tumor slide in 10×magnification. Ki67 positive cells were defined as cells with positive nucleus staining. The Ki67 staining index was defined as the number of Ki67 positive cells divided by the total number of tumor cells in an area on the 60×magnification. Positive and negative controls from human tissue were placed on each slide for validation of the immunohistochemical procedure.

### Statistical Analysis

Comparison of tumor volume between the treatment and control group were calculated using an unpaired student’s t-test. For intra-group comparisons of tracer uptake ANOVA with Bonferroni post-hoc test was used. All data were tested to be normal distributed by means of Kolmogorov-Smirnov test. Correlations between SUVmax ratios and tumor growth were calculated using linear regression. Calculations were made in PASW 17.0 (IBM, Armonk, NY, USA). Data are reported as mean±SEM (unless stated otherwise) and P<0.05 was considered statistically significant.

## Results

### Tumor Growth of APO866 and Vehicle Treated A2780 Xenografts

**Figure 2:**
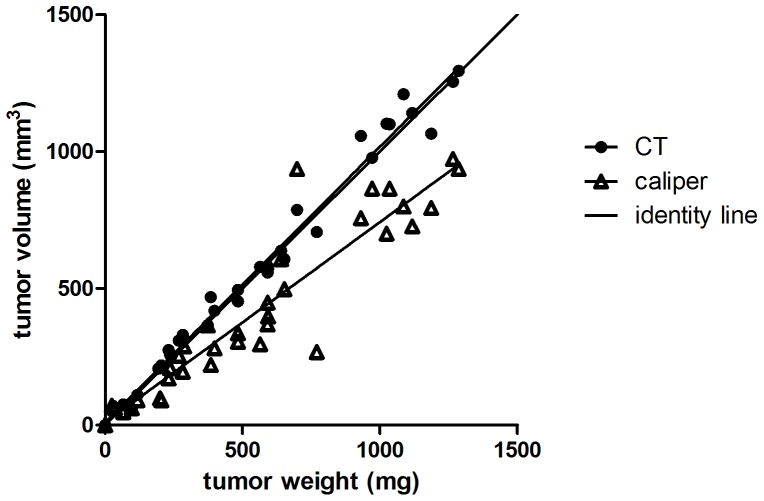
Determination of tumor volume by microCT and caliper. Comparison of tumor weight and tumor volume determined by microCT and caliper respectively (N = 34–35 tumors). An identity line is included in the graph. After the last PET scan the tumors were excised and weighed and tumor weight was correlated with the microCT determined tumor volume measured on Day 7 (P<0.001, r^2^ = 0.98) and caliper determined tumor volume measured on Day 7 (P<0.001; r^2^ = 0.87).

**Figure 3:**
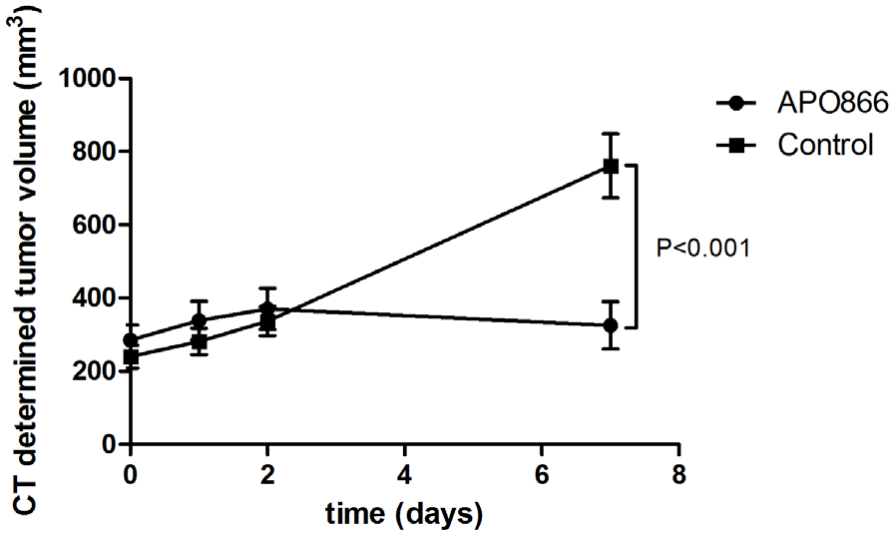
Tumor growth of APO866 and vehicle treated A2780 xenografts. The effects of APO866 on the growth of A2780 tumor xenografts were measured. Tumor volume was determined by microCT. Mice were treated with APO866 (15 mg/kg) or vehicle ip twice daily for 7 days. N = 22 tumors in treatment group and N = 18 in control group. Values are stated as mean±SEM.

Tumor weight correlated with tumor volume determined by microCT (P<0.001; r^2^ = 0.98) and caliper (P<0.001; r^2^ = 0.87) (figure 2). APO866 treatment inhibited growth of A2780 xenograft tumors. Tumors treated with APO866 had volumes (mean±SEM) that were 114±5% (24 h), 128±6% (48 h) and 130±22% (Day 7) relative to baseline volumes at Day 0. In the control group tumor volumes (mean±SEM) were 118±4% (24 h), 145±6% (48 h) and 339±31% (Day 7) relative to baseline volumes Day 0. Tumor volume between the treatment and control group was significantly different at Day 7 (P = 0.001) (figure 3).

### Changes in [18F]FLT and [18F]FDG Following Treatment Initiation with APO866

Baseline levels of [18F]FLT uptake measured as SUVmean were 1.26±0.16 (mean±SD) and SUVmax were 2.18±0.20 (mean±SD). Baseline levels of [18F]FDG uptake measured as SUVmean were 0.67±0.14 (mean±SD) and SUVmax were 1.23±0.29 (mean±SD).

**Figure 4:**
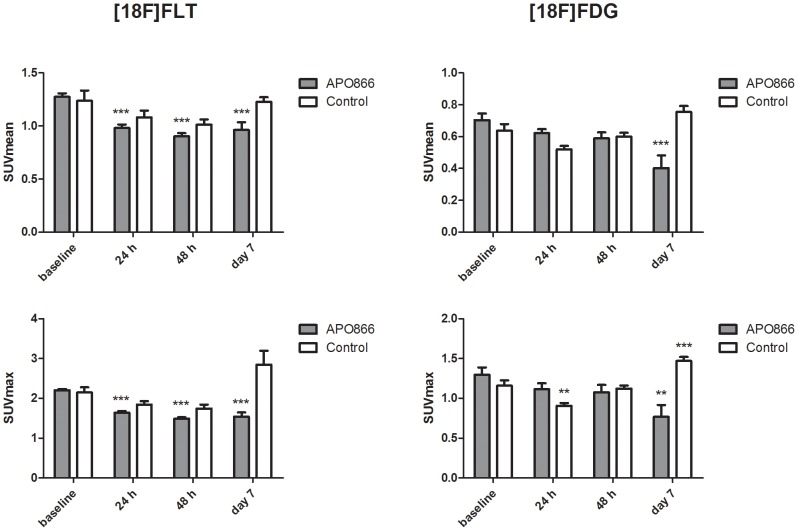
[18F]FLT and [18F]FDG uptake after treatment of A2780 xenografts with APO866. [18F]FLT and [18F]FDG uptake assessed by SUVmean and SUVmax at baseline and following treatment with APO866. Mice were treated with APO866 (15 mg/kg) or vehicle ip twice daily for 7 days. N = 6 tumors in [18F]FLT control group, N = 10 tumors in [18F]FLT treatment group, N = 12 tumors in [18F]FDG control group and N = 12 tumors in [18F]FDG treatment group. The two graphs at left show data from the [18F]FLT experiments and the two graphs at right show data from the [18F]FDG experiments. *) P<0.05, **) P<0.01, ***) P<0.001 versus baseline in same treatment group. Values are stated as mean±SEM.

**Figure 5:**
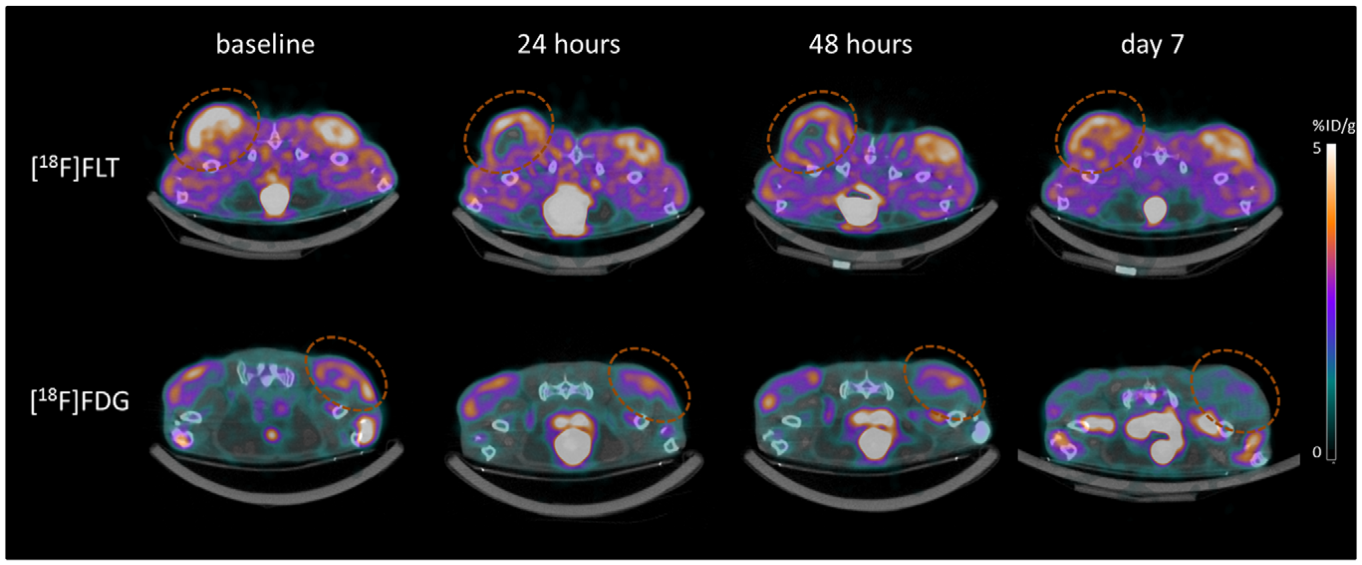
PET/CT images. Combined PET/CT images of a mouse treated with APO866 and scanned with [18F]FLT (upper panel) and a mouse treated with APO866 and scanned with [18F]FDG (lower panel).

Uptake of [18F]FLT measured as SUVmean in the APO866 group was 1.28±0.03 at baseline, 0.98±0.03 at 24 h, 0.90±0.03 at 48 h and 0.96±0.07 at Day 7. Compared to baseline [18F]FLT SUVmean was significantly decreased at 24 h (P<0.001), 48 h (P<0.001) and Day 7 (P<0.001) in the APO866 group. No significant changes in [18F]FLT SUVmean were observed in the control group. [18F]FLT SUVmax uptake in the APO866 group was 2.20±0.03 at baseline, 1.64±0.04 at 24 h, 1.49±0.03 at 48 h and 1.54±0.11 at Day 7. Compared to baseline [18F]FLT SUVmax was significantly different at 24 h (P<0.001), 48 h (P<0.001) and Day 7 (P<0.001) in the APO866 group (figure 4 and 5). No significant changes in [18F]FLT SUVmax were observed in the control group.

Uptake of [18F]FDG measured as SUVmean in the APO866 group was 0.70±0.04 at baseline, 0.62±0.03 at 24 h, 0.59±0.04 at 48 h and 0.40±0.08 at Day 7. Compared to baseline [18F]FDG SUVmean was significantly decreased at Day 7 (P<0.001) in the APO866 group. No significant changes in [18F]FDG SUVmean were observed in the control group. [18F]FDG SUVmax uptake in the APO866 group was 1.29±0.09 at baseline, 1.11±0.07 at 24 h, 1.08±0.09 at 48 h and 0.77±0.15 at Day 7. Compared to baseline [18F]FDG SUVmax was significantly decreased at Day 7 (P = 0.005) in the APO866 group. In the control group [18F]FDG SUVmax was 1.16±0.07 at baseline, 0.91±0.03 at 24 h (P = 0.007 vs. baseline), 1.12±0.04 at 48 h and 1.47±0.05 at Day 7 (P<0.001 vs. baseline) (figure 4 and 5).

**Figure 6:**
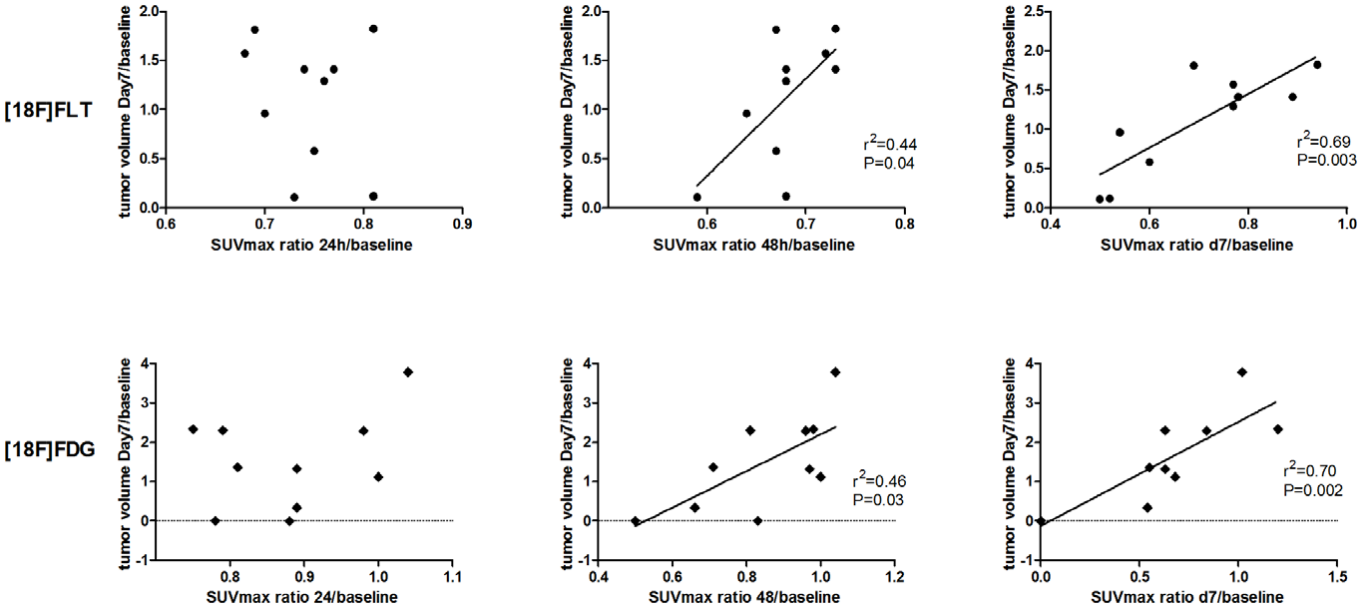
Correlation between SUVmax and tumor growth. Tumor growth assessed by ratio Day 7/baseline compared to uptake of [18F]FLT (upper panels) and [18F]FDG (lower panels) at 24 h (right panels), 48 h (middle panels) and 7 days (left panels). N = 6 tumors in [18F]FLT control group, N = 10 tumors in [18F]FLT treatment group, N = 12 tumors in [18F]FDG control group and N = 12 tumors in [18F]FDG treatment group.

Correlations between SUVmax ratios, from baseline to 24 h, 48 h and Day 7 respectively, and tumor volume changes from baseline to Day 7 for the individual tumors were calculated. For both [18F]FLT and [18F]FDG no significant correlation was found between SUVmax ratio 24 h/0 h and final tumor growth. [18F]FLT SUVmax ratio 48 h/0 h (P = 0.038; r^2^ = 0.44) and d7/d0 (P = 0.003; r^2^ = 0.69) correlated significantly with final tumor growth and [18F]FDG SUVmax ratio 48 h/0 h (P = 0.031; r^2^ = 0.46) and d7/d0 (P = 0.0024; r^2^ = 0.70) correlated significantly with final tumor growth (figure 6).

### Ki67 Immunohistochemistry

**Figure 7:**
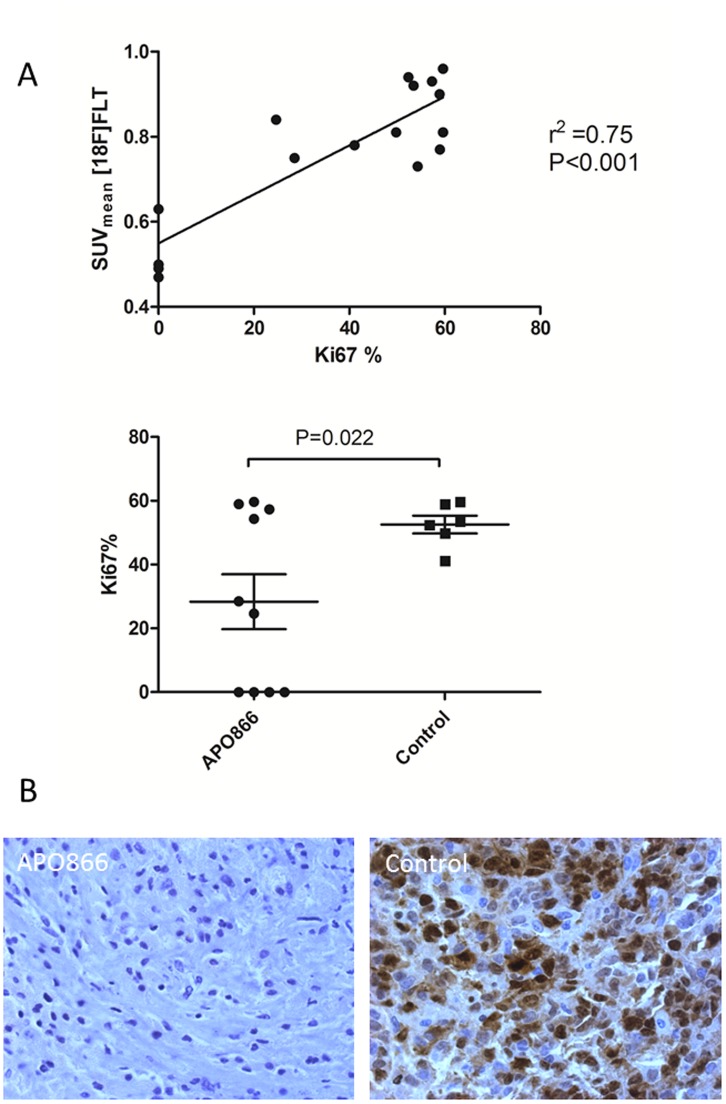
Correlation between Ki67 expression and [18F]FLT uptake. A Correlation of Ki67 protein expression measured by immunohistochemistry with [18F]FLT uptake measured as SUVmean (upper panel). Lower panel compares Ki67 protein expression in the APO866 (N = 10) and control group (N = 6) at the end of the 7 day treatment period. Values are stated as mean±SEM. **B** Representative images of Ki67 stains of an APO866 treated (left) and a control (right) mouse in 40×magnification.

Expression of Ki67 protein measured by immunohistochemistry showed a significant correlation with [18F]FLT SUVmean (r^2^ = 0.75; P<0.001). Differences in expression of Ki67 protein between the treatment (28.3%) and control group (52.5%) were significant (P = 0.022) (figure 7). Some of the treated tumors had 0% Ki67 protein expression. Treatment with APO866 for 7 days resulted in no observable Ki67 positive cells in the slides for these tumors.

## Discussion

In this study we investigated the effect of APO866 treatment on tumor uptake of [18F]FLT and [18F]FDG by PET in the A2780 ovarian cancer tumor model. By groups, uptake of [18F]FLT decreased after initiation of treatment and was significantly different from baseline already at 24 hours after treatment initiation. By groups, uptake of [18F]FDG did not change on the first 1–2 days following treatment start, however at Day 7 uptake was significantly decreased to 50% of baseline uptake. At Day 7 we observed decreases in both [18F]FLT and [18F]FDG tracer uptake. On the PET images (figure 5) it was more easy to see the differences between baseline and Day 7 on the [18F]FDG images than on the [18F]FLT images. [18F]FDG is a more widely available tracer than [18F]FLT and therefore the use of [18F]FDG for detection of later treatment effects on Day 7 can be useful.

A measure of the maximal tracer uptake is often used when assessing treatment responses rather than the mean tracer uptake [28]. With SUVmax the most active metabolic region of the tumor is measured, and one advantage of this is that some of the volume delineation problems often found in SUVmean values is overcome. Much discussion has centered about if inhibiting the most active metabolic or proliferative regions with the anti-cancer treatment is likely to give the best treatment effect. Maximal uptake values are in several studies shown to be a prognostic factor for outcome [28]–[32]. In the present study we found that changes in SUVmax following treatment correlated better with later tumor volume effect than changes in SUVmean. Furthermore, we found a much greater decrease in [18F]FLT SUVmax values at Day 7 compared to SUVmean values.

Lower uptake of tracer at Day 7 compared to baseline for both [18F]FLT and [18F]FDG in the treatment groups was most likely not due to partial volume effect because of a decrease in tumor volume as the tumors did not shrink during the treatment. The changes in tracer uptake were therefore assumed to be due to physiologic changes in the tumor tissue which were further confirmed by the difference in the quantity of Ki67 positive cells between the treatment and control groups. Positive correlation between [18F]FLT uptake and the amount of Ki67 positive cells have earlier been reported by others [33]–[36].

APO866 is a relatively slow acting anti-cancer drug without any immediate cytotoxicity leading to ATP depletion and apoptosis [17], [19]. Since ATP is a co-factor for TK1, a decrease in ATP level will possibly decrease uptake of [18F]FLT, and a change in ATP tumor level is likely to account for some of the [18F]FLT changes we found in this study. This may also explain why we observed earlier changes in [18F]FLT compared to [18F]FDG. Tumor ATP level is decreased 72 hours after treatment start with APO866 [19]. However, already at 24 hours after treatment start a decrease in NAD content was observed. The early change in NAD levels may contribute to the change in [18F]FLT tracer uptake we found already 24 hours after treatment start in this study. No studies have yet analyzed the level of ATP in mouse tumor models following treatment with APO866 and more experiments are accordingly needed in order to elucidate the exact mechanism behind the decreased [18F]FLT and [18F]FDG uptake following treatment with APO866.

Anti-cancer drugs being cytostatic or having low cytotoxicity often require long treatment time before exerting their effect on tumor volume. The advantage of measuring physiological changes as we do in this study with [18F]FLT and [18F]FDG PET compared to the tumor volume criteria is that we are able to measure a treatment effect earlier than we can measure a change in tumor volume.

When the tumors were studied individually, we found a significant positive correlation between the changes in SUVmax for both [18F]FLT and [18F]FDG between baseline and 48 hours and the tumor growth. Accordingly, those tumors which had the greatest decrease in SUVmax values for both [18F]FDG and [18F]FLT 48 hours after treatment initiation were those which had the lowest increase in tumor volume at the end of the experiment. It indicates that we, in addition to finding a decrease in the whole treatment groups, on the individual tumor level already at 48 hours after treatment initiation were able to identify the tumors that responded best to the treatment as it were those that had the greatest change in SUVmax values.

In conclusion, we found a decrease in tumor [18F]FLT uptake following treatment with the nicotinamide phosphoribosyltransferase inhibitor APO866. After 7 days of treatment tumor uptake of [18F]FDG was half of baseline uptake. Additionally, we found that the tumors which responded best to the treatment were those that had the greatest change in both [18F]FLT and [18F]FDG at 48 hours after treatment initiation. These results show the possibility to use [18F]FLT and [18F]FDG to image treatment effect early following treatment with APO866 in future clinical studies.
